# The impact of particulate electron paramagnetic resonance oxygen sensors on fluorodeoxyglucose imaging characteristics detected via positron emission tomography

**DOI:** 10.1038/s41598-021-82754-8

**Published:** 2021-02-24

**Authors:** Philip E. Schaner, Ly-Binh-An Tran, Bassem I. Zaki, Harold M. Swartz, Eugene Demidenko, Benjamin B. Williams, Alan Siegel, Periannan Kuppusamy, Ann Barry Flood, Bernard Gallez

**Affiliations:** 1 Department of Medicine Section of Radiation Oncology, Dartmouth-Hitchcock Medical Center, One Medical Center Drive, Lebanon, NH 03756 USA; 2 Biomedical Magnetic Resonance, Louvain Drug Research Institute, Universite Catholique du Louvain, Brussels, Belgium; 3 Department of Radiology, Geisel School of Medicine, Dartmouth College, Hanover, NH USA; 4 Department of Biomedical Data Science, Geisel School of Medicine, Dartmouth College, Hanover, NH USA; 5 Department of Radiology, Dartmouth-Hitchcock Medical Center, Lebanon, NH USA

**Keywords:** Cancer, Medical research, Oncology

## Abstract

During a first-in-humans clinical trial investigating electron paramagnetic resonance tumor oximetry, a patient injected with the particulate oxygen sensor Printex ink was found to have unexpected fluorodeoxyglucose (FDG) uptake in a dermal nodule via positron emission tomography (PET). This nodule co-localized with the Printex ink injection; biopsy of the area, due to concern for malignancy, revealed findings consistent with ink and an associated inflammatory reaction. Investigations were subsequently performed to assess the impact of oxygen sensors on FDG-PET/CT imaging. A retrospective analysis of three clinical tumor oximetry trials involving two oxygen sensors (charcoal particulates and LiNc-BuO microcrystals) in 22 patients was performed to evaluate FDG imaging characteristics. The impact of clinically used oxygen sensors (carbon black, charcoal particulates, LiNc-BuO microcrystals) on FDG-PET/CT imaging after implantation in rat muscle (n = 12) was investigated. The retrospective review revealed no other patients with FDG avidity associated with particulate sensors. The preclinical investigation found no injected oxygen sensor whose mean standard uptake values differed significantly from sham injections. The risk of a false-positive FDG-PET/CT scan due to oxygen sensors appears low. However, in the right clinical context the potential exists that an associated inflammatory reaction may confound interpretation.

## Introduction

Oxygen is a critical factor in physiology, pathophysiology, and therapy. In many circumstances, the ability to measure oxygen level is critical to assess tissue function and disease^1,2^. Electron paramagnetic resonance (EPR) spectroscopy offers the possibility to provide direct quantitative measurement of the partial pressure of oxygen (pO_2_) in tissues using implanted paramagnetic sensors, whose EPR linewidths are sensitive to the oxygen environment^1,3^. Among the potential EPR oxygen sensors, particulate materials offer several advantages: they are highly sensitive to variations of oxygen (changes smaller than 1 mmHg) and as they are inert in tissues repeated oxygen measurements are possible from the same site over long time periods (months to years)^3^. Several particulate materials currently being used in humans include carbon blacks (also the active component of India ink used in tattoos), charcoals (also used in some inks for marking tissue for long-term follow-up, i.e. in suspicious areas in the colon during colonoscopy), and lithium octa-n-butoxynaphthalocyanine (LiNc-BuO) microcrystals embedded in a biocompatible polymer, polydimethylsiloxane (aka the OxyChip)^4^. Recently, clinical studies in cancer patients have started to demonstrate the feasibility of repeatable measurement of oxygen in tissues using EPR oximetry, with their potential for providing prognostic information and improving outcomes by directing therapy^5^.

The clinical experience with injectable/implantable particulate EPR oximetry sensors in humans is still in its early stages. In total, 71 patients have been injected with carbon particulate sensors. Initial studies with India ink in volunteers totaled fewer than twenty patients each, and established the feasibility of the technique^6^. Tumor oximetry studies using ink particulates at Geisel School of Medicine at Dartmouth/Dartmouth Hitchcock Medical Center (Printex or Carlo Erba ink) have enrolled 27 patients^3^; studies investigating oximetry in human breast tissue using Carlo Erba ink at Emory University reported a total of 9 patients^7^; 19 additional patients received Printex ink injections, most in other types of tumors. A first-in-humans EPR oximetry study using the OxyChip at Geisel School of Medicine at Dartmouth/Dartmouth Hitchcock Medical Center has enrolled over twenty patients (PS, personal communication). As future plans include expansion to larger clinical trials investigating directed hypoxia modification using EPR tumor oximetry, it is expected that more patients undergoing diagnostic imaging in the context of their cancer will have particulate oxygen sensors present.

In July 2017, the investigators involved in a set of clinical EPR oximetry studies that used all three types of particulate materials were alerted to the possible role of an EPR oxygen sensor resulting in a false-positive fluorodeoxyglucose (FDG) positron-emission tomography/computed tomography (PET/CT) examination. The use of the combination metabolic and morphologic PET/CT examination is widely recognized as useful in the management of many types of malignancies^8,9^. Also well recognized is the potential for non-specific metabolic uptake of the FDG due to non-malignant factors, which can lead to false-positive and false-negative results when evaluating malignancies^10^. These imaging findings can result in unnecessary biopsies, additional costs, potential morbidity, and increased worry for patients about disease recurrence. Although our particular interest is in evaluating the oxygen sensors for our clinical studies, there are many medical uses of carbon particulates in common practice including the care of malignancies, e.g., for marking suspicious colon polyps for follow-up, or for marking the exact location of a breast biopsy for future surgery^11^. Additionally, the widespread use of tattoos for cosmetic purposes raises the possible importance for determining whether carbon particulates may lead to artifactual findings on PET/CT imaging.

For these reasons this case prompted further analysis of the potential impact of the injection of EPR oxygen sensors on FDG imaging characteristics in tissues. The present report describes the case, involving a patient on a first-in-humans tumor EPR oximetry trial, in which a false positive FDG-PET/CT scan resulted in an unnecessary biopsy. A retrospective study was then performed investigating all patients enrolled in clinical trials of ink-based oxygen sensors injected into human tumors who also received FDG-PET/CT imaging. A preclinical study investigating the impact of oxygen sensors on FDG-PET imaging is also reported. For the latter study, all four oxygen sensors used in clinical trials (i.e., carbon black particulates [Printex, approved for use at Dartmouth College and Emory University], charcoal particulates [Carlo Erba, approved for use at Dartmouth College, Emory University and West Virginia University; CARBO-REP, approved for use in Belgium], and LiNc-BuO microcrystals embedded in the polymer polydimethylsiloxane [aka “OxyChip”, approved for use at Dartmouth College]) were assessed.

## Materials and methods

### Clinical studies

All procedures performed in studies involving human participants were done in accordance with the ethical standards of the institutional and national research committees and with the 1964 Helsinki declaration and its later amendments or comparable ethical standards. Informed consent was obtained from all subjects. FDG-PET/CT scans were performed on a GE Discovery ST scanner. The dose of FDG was 0.15 mCi/kg, all patients fasted for at least 6 h, serum glucose prior to scanning was less than 250 mg/dl, and scans were performed approximately 60 min after dose administration. The standardized uptake value maximum (SUV_max_) was obtained by defining a region of interest (ROI) that encompassed the lesion, calculating the standardized uptake value (SUV) = (activity concentration in tissue)/(injected activity/body weight), and obtaining the maximum SUV in the ROI.

#### Clinical event

The patient was enrolled in two in vivo oximetry protocols at Dartmouth-Hitchcock Medical Center (DHMC), termed the OxyChip trial (Study 00028499) and the Printex ink trial (Study 00012459), both approved by the Dartmouth Institutional Review Board. The patient had had a prior history of melanoma on the back, but the nodular tumor in the neck being investigated by the studies was determined by pathological examination to be a squamous cell carcinoma. Briefly, following enrollment on the OxyChip trial (National Clinical Trial number NCT02706197), a paramagnetic OxyChip composed of LiNc-BuO was implanted into the patient’s malignant nodule. The pO_2_ surrounding the OxyChip was subsequently measured repeatedly (four measurements, each lasting between 10–30 min, approximately once weekly over four weeks) via non-invasive EPR oximetry, and the OxyChip was then necessarily removed during standard-of-care surgery on the nodule. This protocol was carried out under an investigational device exemption (IDE) for the use of OxyChips, from the Food and Drug Administration. Following surgical removal of the nodule, and after subsequent enrollment in the Printex ink protocol, a paramagnetic sensor material composed of Printex Ink was injected into the post-operative bed prior to therapeutic radiotherapy; non-invasive EPR oximetry was performed repeatedly (nine measurements, each lasting between 10–30 min, approximately weekly over the course of two months) to assess oxygen levels. An IDE is not needed for ink injections (or for EPR oximetry). Per protocol it is not necessary to remove injected ink. This clinical case prompted the investigations described below.

#### Retrospective review

A retrospective review was performed on patients enrolled in two studies investigating EPR oximetry using India Ink at Dartmouth. The retrospective review was approved by the Institutional Review Board at Dartmouth (Study 00031637). The studies reviewed used different inks as paramagnetic sensors; one used Carlo Erba and the other, Printex ink. These studies cover all patients enrolled for tumor oximetry and injected with either ink at Dartmouth. Other studies involving EPR oximetry were not evaluated, due to the required removal of the sensor (OxyChip) or to a low expectation of PET/CT scans at the injection site (foot). Patients enrolled in the two included studies were reviewed for the use of PET/CT imaging performed for re-evaluation of their cancer after ink had been injected. Available images were reviewed for FDG avidity and/or CT findings co-localizing with available photographic images of injected ink (taken on protocol at the time of injection). Co-localizing imaging findings were scored as present or absent.

### Preclinical study

#### Sensor injections

The study was carried out in compliance with the ARRIVE guidelines. Twelve female Sprague–Dawley rats (Janvier, Le Genest-Saint-Isle, France) were used for this study. Animal studies were conducted under an approved protocol (2014/UCL/MD/026) at Catholic University of Louvain, Brussels, Belgium. All applicable institutional and/or national guidelines for the care and use of animals were followed. Animals were divided into 4 groups: (1) CARBO-REP (n = 3): 50 µl of CARBO-REP suspension (40-mg charcoal/ml, Sterylab, Milan, Italy) was administered in the right gastrocnemius muscle using 0.5-ml insulin syringe with 29G needle. The contralateral muscles were assigned for sham injection with needle insertion but no suspension; (2) Carlo Erba (n = 3): 50 µl of charcoal suspension [Carlo Erba (Carlo Erba Reagents, Milano, Italy) 100 mg/ml in saline containing 3% Arabic gum] was administered in the right gastrocnemius muscle of animals using 0.5-ml insulin syringe with 29G needle. The contralateral muscles were assigned for sham injection with needle insertion but no suspension; (3) Printex (n = 3): 50 µl of carbon-black suspension [Printex U (Degussa AG, Frankfurt—Germany), 100 mg/ml in saline containing 3% carboxymethylcellulose] was administered in the right gastrocnemius muscle of animals using 0.5-ml insulin syringe with 29G needle. The contralateral muscles were assigned for sham injection with needle insertion but no suspension; and (4) OxyChip (n = 3): OxyChip was implanted in the right gastrocnemius muscle of animals using a 18G brachytherapy needle. The contralateral muscles were assigned for sham implantation with needle insertion but no OxyChip. The suspensions used (Carlo Erba, Printex, and CARBO-REP) are identical to those used in humans. The OxyChip implanted is identical to that used in humans. The injection protocol for all four particulates used the same standard methods as for humans. All implantations were performed under aseptic conditions while rats were anesthetized with a mixed solution of ketamine and xylazine (doses of 80 and 10 mg/kg, respectively).

#### Micro-PET imaging of rats

Micro-PET imaging was carried out at 4, 47, 97, and 181 days after oxygen-sensor implantation. Before every PET scan, animals were fasted overnight. FDG (Betaplus Pharma, Brussels, Belgium) was injected intraperitoneally with a dose of 400–600 µCi. PET acquisitions were performed 1 h after tracer injection on a dedicated small-animal PET scanner (MOSAIC, Philips) with a spatial resolution of 2.5 mm (FWHM). Rats anesthetized with 2% isoflurane underwent first a 10-min emission scan followed by a 10-min transmission scan using a 370-MBq 137Cs source^12^. After the correction with attenuation factors obtained from the transmission scan, images were reconstructed using a fully 3D iterative algorithm (3D-RAMLA) in a 128 × 128 × 120 matrix, with a voxel of 1 mm^3^. Regions of interest (ROI) were delineated using PMOD software (PMOD Technologies Ltd, Zurich, Switzerland). The 2D ROIs were established on consecutive transversal slices using a 30% iso-contour tool that semi-automatically created a 3D volume of interest (VOI) encircling the tissue of interest. The result of tracer uptake was expressed as SUV means, calculated as the FDG uptake normalized to injected dose and body weight of animals.

#### Statistics and data analysis

The two-way analysis of variance (ANOVA) was performed for each sensor as shown in Fig. 3^13^. The null hypothesis was that the response curve across time of the FDG signal in Sensor and Sham groups was the same. All calculations were carried out in statistical package R [(R Core Team (2019). R: A language and environment for statistical computing. R Foundation for Statistical Computing, Vienna, Austria].

## Results

### Clinical event

**Figure 1:**
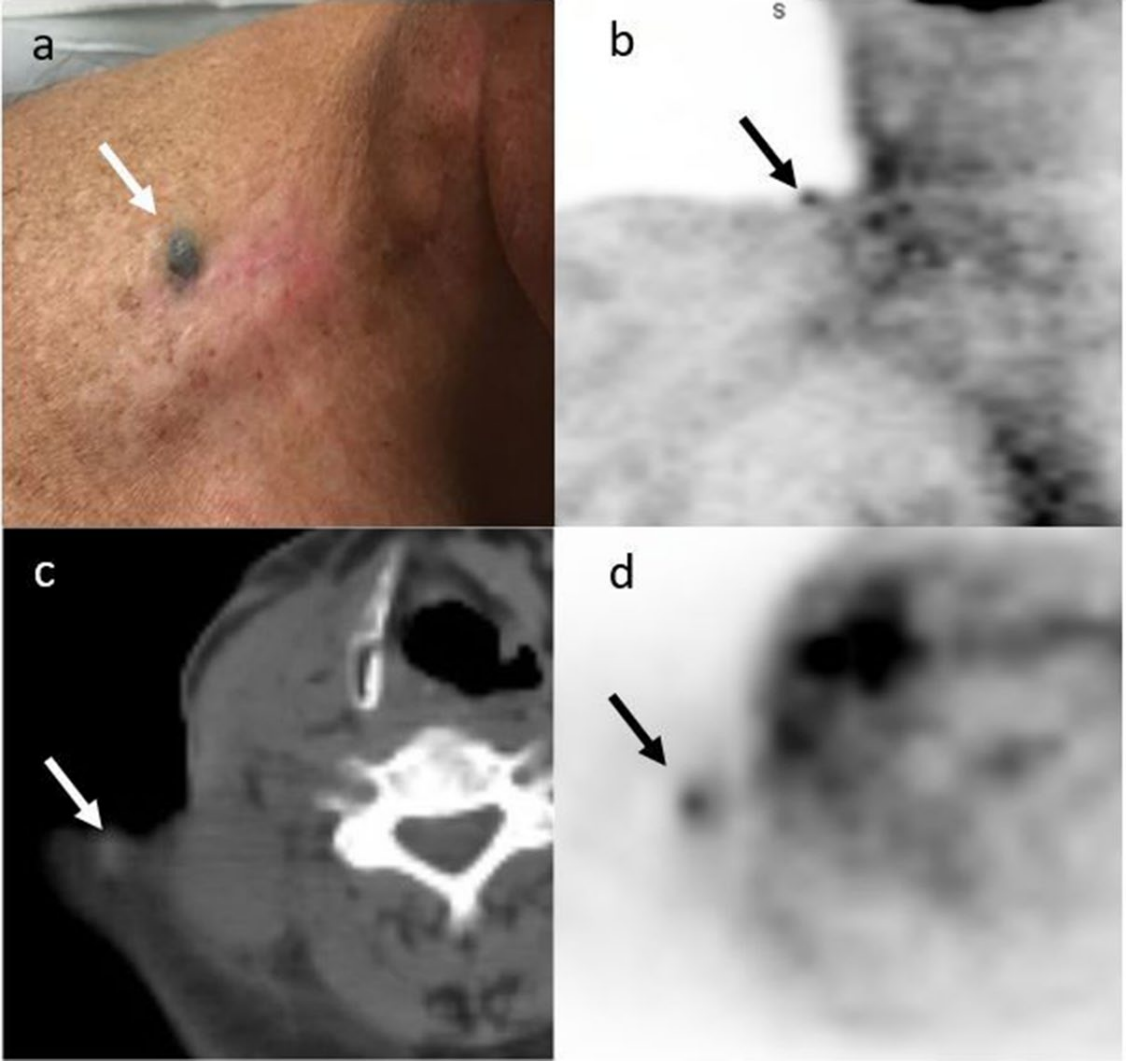
(**a**) Printex ink injected into subcutaneous tissue of the postoperative bed (white arrow). (**b**) Coronal PET image through ink injection demonstrating FDG avid lesion (black arrow). (**c**) On the axial CT component of the PET-CT, a focally dense area is evident corresponding to the region of the injected ink (white arrow). (**d**) Registered PET image to CT from (**c**). The area noted on the CT is FDG avid (black arrow).

The patient, a 62-year-old male, presented in 2016 with irregular pigmentation on his right mid back. A wide local excision and sentinel lymph biopsy demonstrated a pT3bN3 (Stage IIIC) melanoma with three positive nodes. A restaging PET/CT scan (prior to participating in the oximetry studies) following the surgery revealed a new hypermetabolic right lower cervical lymph node. He was scheduled for surgical removal of this lymph node and consented to participate in the OxyChip clinical study. He was implanted with an OxyChip, underwent successful measurements, and had his scheduled surgery (which simultaneously removed his OxyChip). The pathology report unexpectedly revealed that the lymph node was a squamous cell carcinoma (SCC); no primary SCC malignancy was found. Adjuvant radiation therapy to the post-operative bed, for a total of 66 Gy in 33 fractions, was recommended. Immediately prior to beginning his radiotherapy the patient consented to participate in a second oximetry study, and Printex ink was injected into the post-operative bed (Fig. 1a). Nine successful EPR oximetry measurements were subsequently made^5^. In keeping with the study protocol, the Printex ink was not removed.

Approximately 25 weeks after the Printex injection and 16 weeks after completion of radiation therapy, the patient returned for a routine follow-up visit. In a physical examination, the physician reported that “the lesion where the Printex ink was placed appears to be more prominent and elevated”. A reading of a PET/CT scan from the same day reported “A 6 mm FDG avid cutaneous lesion in the medial right shoulder, corresponding to a lesion noted on recent dermatology examination, suspicious for malignancy. An inflammatory etiology is not excluded. No evidence of residual active disease in the neck and no evidence for distant metastatic disease.” The SUV_max_ was calculated at 1.57. See Fig. 1b–d.

An excisional biopsy was performed on the same day. Pathology revealed “dense nodular deposition of black-to-brown pigment in the dermis associated with pigment-laden macrophages and focal dermal scar. The findings are consistent with tattoo in the appropriate clinical setting.” The case was discussed with the patient’s oncologist and no further treatment was recommended at that time. As of the time of this report, the patient has had no evidence of a local recurrence in the head and neck region, although he did develop a subsequent lung metastasis.

The study investigators reported this incident to the Dartmouth Institutional Review Board immediately. Six types of actions were taken in response to this case: (1) All participants in the ink studies were notified that, if they were to have an FDG-PET/CT scan involving the region with the ink, they may experience a false-positive reading and therefore should bring their ‘tattoo’ to the attention of their treating physician and (2) this was added as a potential risk to the written informed consent. (3) The investigators on all studies involving oximetry at DHMC or other institutions were notified about this incident and the possibility of a false positive reading on an FDG-PET/CT and asked if they had been aware of any other cases (they had not). (4) A review of the literature was conducted regarding the circumstances surrounding positive and false-positive rates for FDG-PET/CT scans and whether India ink ‘tattoos’, carbon particulates, or EPR oxygen-sensing devices may be related to an increased risk of false positives. (5) A retrospective chart review of appropriate ink patients in Dartmouth’s clinical studies was performed to search for other instances of an FDG-PET/CT in patients injected with ink. (6) The investigators in Brussels initiated a preclinical investigation to learn more about whether the hypermetabolic activity on the scan appeared to be time-dependent and/or sensor-dependent. Results from the last two efforts are reported below.

### Retrospective chart review

All patients participating in EPR tumor oximetry studies using Printex or Carlo Erba ink at DHMC were included in the retrospective chart review. In addition to the case report described above a total of 22 patients were eligible for review (19 Printex patients, and 3 Carlo Erba patients). No Carlo Erba patient had a follow-up FDG-PET/CT with ink present. Six Printex patients had FDG-PET/CT exams following injection. Of these, two were not relevant to this review (one had had the ink removed in a cancer surgery conducted prior to the FDG-PET/CT and the other’s FDG-PET/CT did not include the area of the ink). Of the remaining four, three had no suspicious nodules on the CT, or FDG avidity above background, corresponding to the areas of ink injection. These three patients had ink injected into different areas: the first was into the skin overlying the right groin, the second was into the skin overlying the stump of his remaining leg after an amputation, and the third was into the skin overlying the scapula. A total of seven FDG-PET/CTs were performed in follow-up for these three patients. One patient with a squamous cell carcinoma of the pyriform sinus had FDG avidity in the area of ink injection (injected into a cervical lymph node). The FDG-PET/CT scan was performed 5 months after injection (and 3 months after definitive chemoradiotherapy) and revealed persistent nodal disease as well as distant metastases, including areas of FDG avidity in the injected lymph node. In the context of active cancer in the area of ink injection no definitive conclusions can be drawn regarding whether FDG avidity was associated with the ink. However, due to the extent and depth of FDG avidity in the node it was felt by the physicians performing the review (Dr. Siegel and Dr. Schaner) unlikely to be related to the superficially injected ink.

### Preclinical study

**Figure 2:**
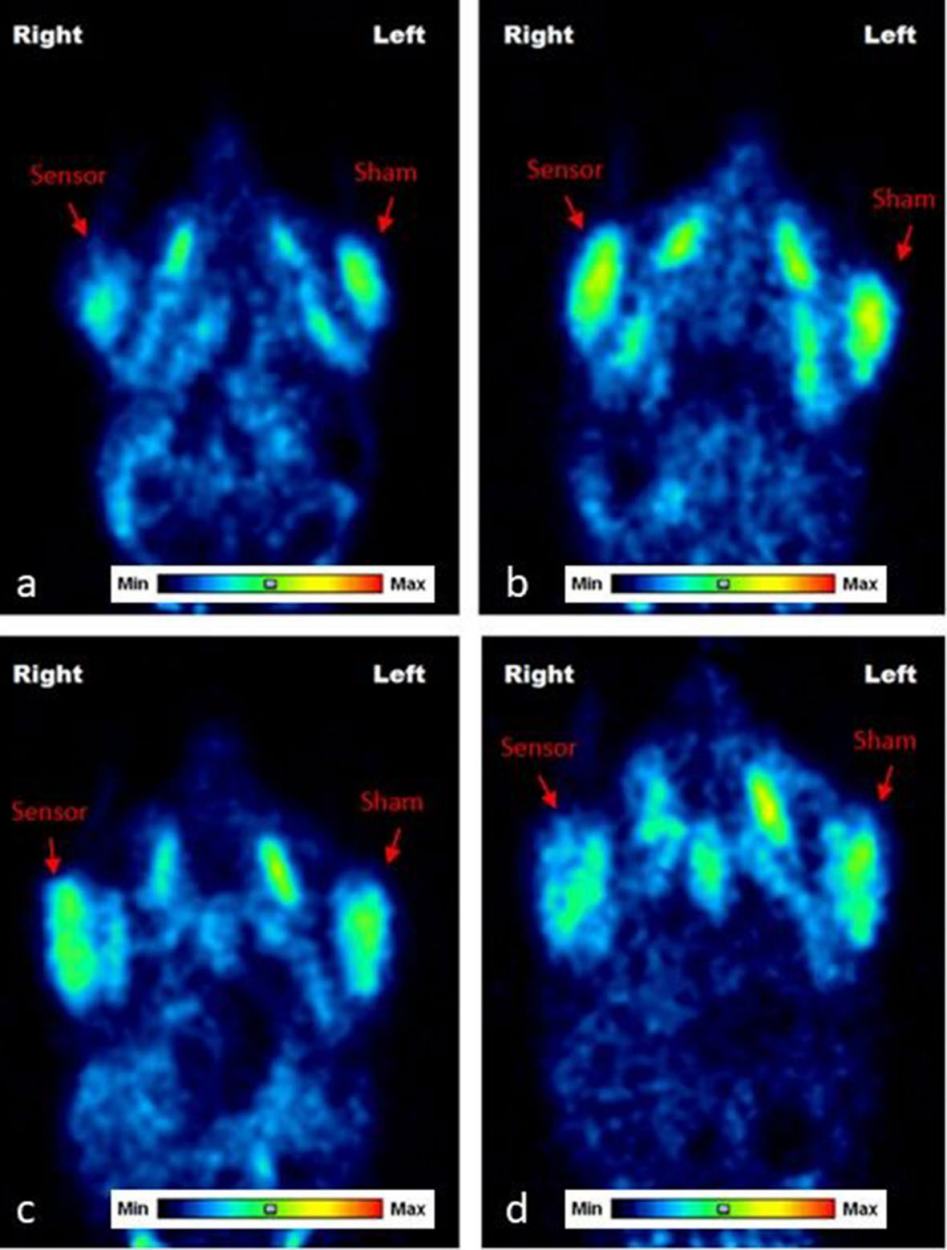
Representative PET images illustrating FDG imaging characteristics in a rat injected with Printex oxygen sensor in the right gastrocnemius muscle and sham injection in the left one. The PET images were acquired at (**a**) 4 days, (**b**) 47 days, (**c**) 97 days, and (**d**) 181 days. Images were normalized to a common background intensity. Each image is an independent activity-level range with the same intensity of background. No quantitative intercomparison should be made between images. The images provide a qualitative comparison of relative intensity between a region of interest and other portions of a given animal's tissue.

**Figure 3:**
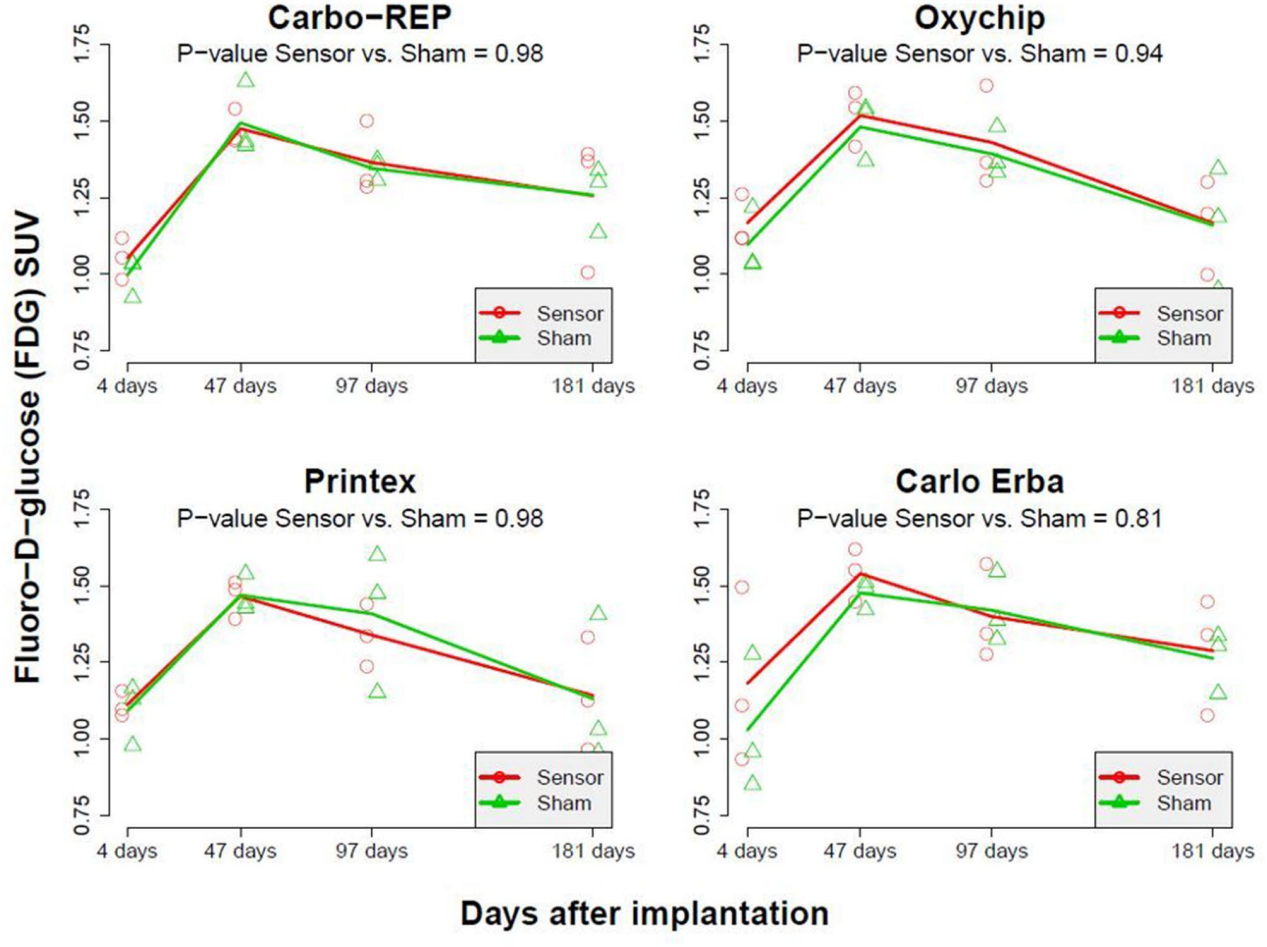
FDG SUV mean values for sham and sensor in rat muscles as measured by PET at 4, 47, 97 and 181 days after sensor administration. For all four types of oxygen sensors no significant difference in FDG SUV mean was found between the sensor-implanted muscles and sham muscles across all time points.

Figure 2 shows typical micro-PET images recorded at days 4, 47, 97, and 181 days after the administration of Printex, the oxygen sensor that was at the origin of the clinical case report. The accumulation of FDG was similar in both muscles (the muscle injected with the sensor and the sham injected muscle); data are presented in Supplementary Table 1. The analysis of FDG SUV mean was similar for all groups of rats. As shown in Fig. 3, there were no significant differences in FDG SUV means between sensor-injected muscles and sham-injected muscles, whatever the oxygen-sensor used (CARBO-REP *p* = 0.98, OxyChip *p* = 0.94, Printex *p* = 0.98, Carlo Erba *p* = 0.81). This again suggests that there was no evidence that false positives would be commonly expected for the oximetry sensors or carbon particulates in general.

Of note, there appeared to be a trend for the SUV mean to increase at 6 weeks compared to the mean at the beginning of imaging, and then for the mean to decrease over the remaining several months. This is consistent with the aging of the animals^14^. Of particular note for this study, these trends occurred consistently in all sensors *and* in all sham injections, i.e., it was not linked to presence of the oxygen sensor in the muscle.

## Discussion

FDG-PET/CT imaging has been in use clinically for more than 25 years and is one of the fastest growing technologies in nuclear medicine. It is used to diagnose, stage, and restage many types of malignancies including melanoma and head and neck cancer^9,10,15–17^. The current investigation stemmed from one case, in which a patient with both melanoma and head and neck SCC participating in clinical oximetry studies had an FDG-avid lesion felt to be concerning for recurrence. It was biopsied but was determined in fact to be a false-positive PET finding, presumably related to his ink injection. This prompted an investigation into the impact of clinical EPR oximetry sensors on FDG-PET/CT imaging outcomes.

The preclinical data presented here demonstrated no evidence that any of the four clinically used EPR oximetry sensors investigated increase FDG avidity when injected into rat muscle. Importantly, the oxygen sensors and placement techniques used in these experiments were identical to those approved for human use. There was no difference between the FDG reading for any sensor compared to the sham injection, at any of the four time points measured, up to six months. These data suggest that other factors, beyond the sensor in and of itself, contributed to the false-positive FDG-PET/CT in this patient.

A retrospective analysis of 22 patients enrolled at Dartmouth in two tumor EPR oximetry studies injected with Printex or Carlo Erba ink yielded only four patients who met the inclusion criteria for analysis, all of whom were injected with Printex ink. Of these, none had compelling evidence of FDG avidity associated with the ink injection.

Although clinical oximetry studies use three different types of particulates (carbon black, charcoal, and LiNc-BuO) this case involved carbon black, which is the same ingredient commonly used in black tattooing and black medical markings, particularly in the US. Therefore, in addition to investigating clinical oximetry sensors, we wanted to investigate whether there was any evidence in the literature that black tattooing or medical markings were implicated in any false positive readings for cancer patients. FDG is not tumor-specific in its identification of glucose consumption and therefore there is a significant potential for avid areas being associated with other factors, which can result in false-positive identification of malignancies^18–24^. False-positives have been widely reported secondary to factors ranging from the injection of imaging or contrast agents, differences in healthy and diseased tissues that cause variations in FDG uptake, artifacts related to the presence of metal and other objects in tissues, and due to inflammatory and other responses to injury (particularly when the test is used for restaging following surgery, chemotherapy, and/or radiotherapy where up to 40% of the agent may be taken up by non-tumor tissue). For these reasons an interim of 12–16 weeks after completion of therapy before imaging with PET/CT is usually recommended^10^. Concurrent diseases can also impact the uptake of the FDG including diabetes, tuberculosis, sarcoidosis and autoimmune diseases. Age, body mass and factors such as variation in the amount of the imaging reagent can also impact the images, and some advocate use of standardized uptake values in order to normalize and minimize any impact from these variations^24^. Patient activities at the time of imaging can cause some variations in uptake of the agents, with recommendations regarding patient care and history taking prior to imaging to minimize these artifacts^25^. We found no articles talking about India ink tattoos or medical markers and FDG uptake; one false-positive reading in a lung that was apparently related to carbon particulates was reported in a woman who had chronic exposure to wood burning^26^. However, there is typically a mild inflammatory-like response in the body’s reaction to tattoos and markers (which generally involve a typical reaction to inert foreign body materials with macrophage cells surrounding the particulates), and macrophages typically are permanently present at most or all sites of India ink markings^27^. This finding is consistent with the histopathology found in this patient. Inflammatory processes are well known to be potential causes of false-positive readings, and it is likely that this reaction, rather than the ink in and of itself, resulted in the FDG avidity associated with the lesion^20–22^.

It is important to note that the injection of particulate inks for clinical EPR oximetry results in variable ink deposition, ranging from a focused concentration of ink to a much more diffuse pattern of distribution. A constellation of findings in the patient likely led to a biopsy and subsequent confirmation of a false positive. Firstly, the ink coalesced into a firm bleb (perhaps in the context of ink injection into a post-operative bed) that was subsequently evident on the CT component of the FDG-PET/CT imaging and led to concerns regarding a dermal tumor deposit. Secondly, the patient had had a previous diagnosis of melanoma, raising concern for skin metastases, and the dark color of the ink confounded the physicians’ ability to clinically distinguish a recurrence from ink. Thirdly, the injected ink appears to have generated an inflammatory response sufficient to result in clinically identifiable FDG avidity. Lastly, the radiologist reading the scan was aware of the clinical skin finding and melanoma diagnosis, calling attention to a lesion that may have otherwise been interpreted as more likely benign given its size and relatively low SUV_max_ of 1.57.

## Conclusions

In the patient event reported here, the confluence of an ink injection associated with an inflammatory response, FDG avidity associated with an area of increased density on CT imaging, and clinical concerns about a dark lesion in the context of an advanced melanoma on the back, appears to have led to an unnecessary biopsy. The data presented support a low risk for a false positive finding using FDG-PET/CT imaging due to the oximetry sensors evaluated. However, as ink particulates are increasingly being used in medical applications, it will be important to be aware that, although unlikely, in certain circumstances they could contribute to a false-positive finding on an FDG-PET/CT scan. This FDG avidity is likely secondary to an associated inflammatory response. Awareness of the clinical context (including involvement in research studies) and discussion with providers is critical in order to make appropriate clinical decisions.

## Supplementary Information

Supplementary Information 1.

Supplementary Information 2.

## Data Availability

Authors will make materials, de-identified data and associated protocols promptly available to readers without undue qualifications in material transfer agreements.
