# Supplementary material for: The impact of particulate electron paramagnetic resonance oxygen sensors on fluorodeoxyglucose imaging characteristics detected via positron emission tomography
Source: Sci Rep. 2021 Feb 24;11:4422. doi: 10.1038/s41598-021-82754-8 (PMC7904945; doi:10.1038/s41598-021-82754-8)
Supplement: Supplementary file 2 — Supplementary Information 2. [file 41598_2021_82754_MOESM2_ESM.docx]

**Legend:**

**Supplementary Table 1:** micro-PET data for CARBO-REP, OxyChip, Printex, and Carlo Erba sensors after injection into rats. Each sensor was injected into three rats; the sensor was injected into the right gastrocnemius muscle, and a corresponding sham injection occurred in the left gastrocnemius muscle (n=12). Each rat was imaged at day 4, 47, 97, and 181 after injection. For each time point the mean standard uptake value (SUV) measurements are reported for both sensor and sham, as well as the mean SUV of the three respective measurements.
